# Role of Dendritic Cells in Natural Immune Control of HIV-1 Infection

**DOI:** 10.3389/fimmu.2019.01306

**Published:** 2019-06-06

**Authors:** Enrique Martin-Gayo, Xu G. Yu

**Affiliations:** ^1^Hospital Universitario de la Princesa, Universidad Autónoma de Madrid, Madrid, Spain; ^2^Ragon Institute of MGH, MIT, and Harvard, Massachusetts General Hospital, Harvard Medical School, Boston, MA, United States

**Keywords:** dendritic cell, HIV-1 controller, IFN, Tfh, bNAb, vaccine

## Abstract

Dendritic cells (DCs) are professional antigen-presenting cells that link innate and adaptive immunity and are critical for the induction of protective immune responses against pathogens. Proportions of these cells are markedly decreased in the blood of untreated HIV-1-infected individuals, suggesting they might be intrinsically involved in HIV-1 pathogenesis. However, despite several decades of active research, the precise role and contribution of these cells to protective or detrimental host responses against HIV-1 are still remarkably unclear. Recent studies have shown that DCs possess a fine-tuned machinery to recognize HIV-1 replication products through a variety of innate pathogen sensing mechanisms, which may be instrumental for generating both cellular and humoral protective immune responses in persons who naturally control HIV-1 replication. Yet, dysregulated and abnormal activation of DCs might also contribute to sustained inflammation and immune activation accelerating disease progression during chronic progressive infection. Emerging data also suggest that DCs can influence the induction of potent broadly-neutralizing antibodies, and may, for this reason, have to be considered as important components of future HIV-1 vaccination strategies. Apart from their involvement in antiviral host immunity, at least a subgroup of DCs seem intrinsically susceptible to HIV-1 infection and may serve as a viral target cell population. Indeed recent studies suggest that specific DC subpopulations residing in the genital mucosa are preferentially infected by HIV-1 and play an active role in sexual transmission; therefore, DCs may contribute to viral dissemination and possible persistence of the viral reservoirs through either direct or indirect mechanisms. Here, we analyze the distinct and partially opposing roles of DCs during HIV-1 disease pathogenesis, with a focus on implications of DC biology natural immune control and HIV cure research efforts.

## Introduction

Dendritic cells (DCs) represent a heterogeneous family of immune cells that link innate and adaptive immunity. The main function of these innate cells is to capture, process, and present antigens to adaptive immune cells and mediate their polarization into effector cells (1). DCs can be subdivided in two main subtypes: plasmacytoid (pDC) and myeloid (mDC) DCs, which specialize in the recognition of different pathogen associated molecular patterns (PAMPs) due to the unique distribution of Pattern Recognition Receptors (PRR), such as toll-like receptors, C-type lectins and intracellular nucleic acid sensors (2–4). As a result, mDCs and pDCs can efficiently induce CD4^+^ and CD8^+^ T cell responses against different types of pathogens. In addition, both mDCs and pDCs are also capable of interacting with Natural Killer (NK) cells, which are particularly relevant during viral infections (5). Therefore, the contribution of different DC subtypes to immune responses against microbial infections seems to be highly complex and be influenced by context- and pathogen-dependent factors.

During HIV-1 infection, several effector components of the innate and adaptive immune system are involved in the host antiviral response, and although these immune responses seem unable to prevent the establishment of the infection, they can influence HIV-1 disease progression. Effective immune control of HIV-1 infection occurs in rare population of HIV-1 infected individuals who are able to spontaneously control HIV-1 replication in the absence of antiretroviral therapy, and to maintain undetectable levels of viral replication as measured by commercial PCR assays. In these individuals, long-lived polyfunctional HIV-1-specific CD8^+^ T cells have been identified as the main biological correlate of spontaneous immune control of HIV-1 (6–8). However, the contribution of DCs to durable immune control of HIV-1 is still a relatively unexplored area and a matter of active debate. During the last years, new relevant data about DC biology in the context of HIV-1 infection have become available, specifically with regards to DC susceptibility to infection, to DC-mediated immune regulation and to direct host-pathogen interactions between DC and HIV-1. In this review, we have focused on consolidating the most recent advances on DC biology in the context of HIV-1 immunopathology, and on providing a detailed evaluation of the role of DC in HIV-1 immune control.

## Anatomical Localization and Activation of DCs During HIV/SIV Infection

DCs are physiologically distributed in mucosal and lymphoid tissues where they capture antigens and present them to T cells, but a small proportion of mDCs and pDCs are also circulating in the blood. mDCs can be identified as lineage marker negative cells that display high surface levels of CD11c and HLA-DR (9) while pDCs are CD11c^−^HLA-DR^+^ cells characterized by surface expression of the C-type lectin BDCA2, high levels of the alpha chain of the receptor for interleukin-3 (CD123) and the immunoglobulin superfamily receptor immunoglobulin-like transcript 7 (ILT7) (10). Upon HIV-1 infection, the anatomical distribution of DCs is dramatically altered and lower proportions of pDCs and mDCs are present in the blood of infected untreated individuals (11–13). The extent of the depletion of circulating mDC is correlated with rapid disease progression during HIV-1 and SIV infections (14, 15). Interestingly, proportions of circulating pDCs are more profoundly reduced in HIV-1 progressors in contrast to controllers (16); although the exact mechanisms responsible for these differences remain unknown. Despite these discrepancies, circulating pDCs from both controllers and progressors are characterized by upregulated expression of the gut homing integrin α4β7, suggesting selective trafficking to mucosal intestinal tissue where the majority of HIV-1-infected cells reside (17). Similarly, higher levels of activation in gut resident mDCs and pDCs seem to be associated with changes in gut microbiota and immune homeostasis (18). In addition to migration to the gut, preferential recruitment of pDCs to the lymph nodes also occurs in HIV-1-infected subjects (19).

Besides the changes in anatomical distribution, circulating and tissue-resident DCs display an activated phenotype defined by upregulation of costimulatory molecules in infected individuals (11–13). In fact, higher levels of activation in blood DCs seem to correlate with plasma viremia in progressors (20). In contrast, less pronounced phenotypical signs of immune activation, combined with increased functionality have been described in mDCs from the blood of HIV controllers (21). Interestingly, highly activated mDCs residing in the lymph nodes from HIV-1^+^ patients seem to co-express inhibitory costimulatory molecules such as PD-L1 and are still capable of responding to TLR stimulation, in contrast to cells from peripheral blood (19).

**Figure 1:**
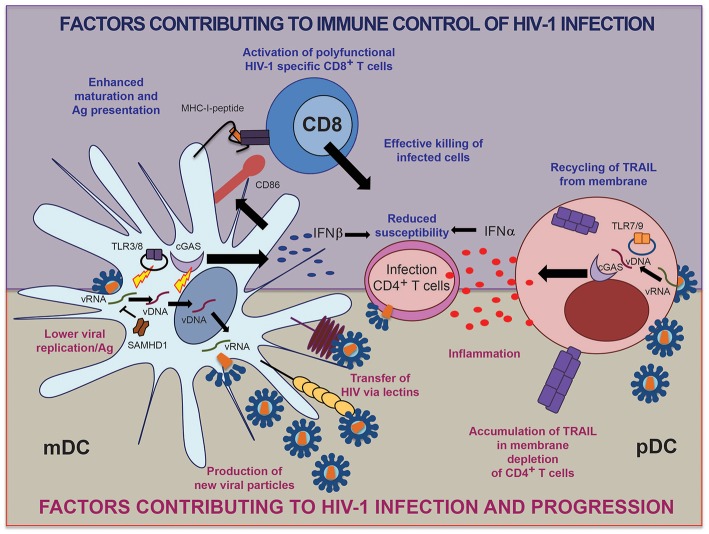
Schematic representation of factors in human mDC and pDC contributing to immune control vs. progression of HIV-1 infection.

A hallmark of circulating pDCs from the blood of HIV-1^+^ individuals is the expression of high basal levels of type I interferon (IFN) and IFN-stimulated genes, likely reflecting abnormal immune activation (22). Interestingly, this higher baseline activation of IFN-dependent immune activity seems to make pDCs from progressors refractory to antigenic stimulation (23, 24), and paradoxically reduces their ability to secrete appropriate levels of IFN-α upon PRR stimulation. In contrast, pDCs from controllers maintain IFN-α secretion levels that are comparable to those of healthy individuals. Consistent with these findings, microscopy-based studies indicated differences in the trafficking of intracellular TNF-related apoptosis-inducing ligand (TRAIL) in pDCs from controllers and healthy donors compared to progressors. TRAIL is a molecule known to induce apoptosis of CD4^+^ T cells through a mechanism regulated by the alarmin High Mobility Group Box 1 (HMGB1) (23, 25). While TRAIL seems to be recycled from the membrane of pDCs in controllers after exposure to HIV-1, pDCs from viremic patients appear to constitutively express TRAIL on the membrane, which may contribute to unspecific induction of cell death in CD4^+^ T cells and accelerate cell loss and immunodeficiency (26) (Figure 1). Overall, these data indicate that pDCs from controllers maintain a functional profile that is similar to healthy persons. In contrast, pDCs from progressors exhibit a hyperactivated state characterized by constitutive TRAIL up-regulation, higher basal levels of IFN-dependent immune responses, and a reduced ability to produce IFN-α in responses to antigen exposure, most likely as a result of generalized immune activation that makes cells refractory to microbial stimulation (Figure 1). The normal functional profile of pDCs in controllers therefore could be a consequence, rather than a cause of viral immune control. Notably, the initiation of antiretroviral therapy does not revert the decline in pDC frequency and function observed during progressive infection, suggesting an irreversible defect in pDC physiology in progressors after prolonged exposure to high viremia (27). Interestingly, less pathogenic HIV-2 strains induce lower levels of type I IFN expression in pDCs compared to HIV-1, suggesting that lower levels of pDC activation could be associated with immune control of the infection (28). In addition to pDCs, recent works in the SIV model have suggested that additional cell types might be responsible for abnormal activation of type I IFN responses at later stages of progressive infection (29). Together, cumulative information from recent studies suggests that DC distribution and function might be critically altered during HIV-1 infection and that preservation of physiological DC distribution and function is associated with immune control of the infection.

## DCs as Vehicles for HIV-1 Transmission and Dissemination

During the last few years, several studies have shown that DCs have the ability to transfer HIV-1 particles to target CD4^+^ T cells and facilitate their infection, in a process known as trans-infection (30). This phenomenon starts with the transference of HIV-1 virions to pockets in the membrane of DCs, where they accumulate and are subsequently actively transferred to T cells through virological synapses (31). The stability of such transmission events depends on the expression of adhesion molecules, such as Intercellular Adhesion Molecule (ICAM) (32) and the actin assembly machinery (33). In order to transfer viruses to CD4^+^ T cells, DCs require the capture of HIV-1 particles through the lectin Dendritic Cell Intercellular Adhesion Molecule-3-Grabbing Non-integrin (DC-SIGN) (34, 35), and the Sialic acid-binding Immunoglobulin-type Lectin 1 (SIGLEC-1) receptor (36). Recent reports have shown that the ability of DCs to facilitate HIV-1 trans-infection is acquired upon activation with inflammatory molecules associated with poor HIV-1 prognosis, such as IFNα and LPS (37). These stimuli have been shown to induce SIGLEC-1 expression and therefore, enhance the capture and transfer of viral particles. Notably, circulating mDCs but not pDCs facilitate trans-infection of HIV-1 (32). Therefore, increased basal levels of immune activation and high-level viremia might contribute to disease progression through facilitation of viral trans-infection by mDCs. Consistent with a role of mDCs in supporting HIV-1 trans-infection in lymphoid tissues, it was shown that depletion of lymph node-resident mDCs in tissue-suspension cultures reduced the efficiency of HIV-1 infection of CD4^+^ T cells (38). However, mutations of SIGLEC-1, which naturally occur in small proportions of individuals, did not seem to provide protection from HIV-1 infection or attenuation of HIV-1 disease progression (39), suggesting that classical, SIGLEC-1-independent HIV-1 dissemination within in the host remains the predominant mechanisms fueling viral infection *in vivo*.

## DC Susceptibility to HIV-1 Infection and Host Restriction Factors

Most DCs express the coreceptor CD4 (40), and therefore are in principle susceptible to infection with HIV-1. However, DCs seem to represent a more hostile and restrictive environment for HIV-1 than CD4^+^ T cells, for reasons that are not completely clear. While initial studies suggested that monocyte derived DCs (MDDCs) are highly resistant to infection with HIV-1 (41), primary mDCs are able to support some levels of HIV-1 replication, at least *in vitro* (42–44).

The main restriction factor that limits HIV-1 replication in MDDCs and mDCs seems to be the cytoplasmic protein SAM domain and HD domain-containing protein 1 (SAMHD1), which is highly expressed in myeloid cells and is able to block HIV-1 replication at the retro-transcription level by depleting endogenous intracellular pools of dNTPs (45), and by directly degrading viral RNA (46). While it is clear that SAMHD1 is a key factor limiting replication of HIV-1 in MDDCs (47) and inhibiting further spread of virions to T cells (48), recent studies demonstrated that MDDCs can actually support productive infection with HIV-1 to a certain degree, despite high levels of expression of this restriction factor (49). The functional ability of SAMHD1 to restrict HIV-1 replication is regulated by phosphorylation mediated by host kinases from the cyclin-dependent kinase family (50). Interestingly, the functionally active, de-phosphorylated form of SAMHD1 is preferentially found in primary DCs isolated *ex vivo* from human blood, which potentially could contribute to a higher resistance to infection (51). However, it is unclear whether restriction of HIV-1 by SAMHD1 in mDCs might truly benefit the host, since restriction of HIV-1 replication via SAMHD1 may impair cytoplasmic viral immune recognition in mDCs and impair their ability to prime HIV-1-specific T cells. On the other hand, interactions of mDCs with T cells induce downregulation of SAMHD1 expression (52), allowing human primary mDCs to be more permissive to infection (44). Importantly, recent data indicate that primary CD1c^+^ and CD141^+^ mDC subtypes might differ in their susceptibility to HIV-1 infection. In this regard, expression of the endosomal protein RAB15 prevents fusion of viral particles in CD141^+^ mDCs and induces a higher level of cell-intrinsic resistance to infection with HIV-1 and HIV-2 compared to CD1c^+^ mDCs (53). Further proof for the susceptibility of primary mDCs to HIV-1 infection was provided by a recent study identifying a distinct population of CD1a^+^ mDCs residing in the vaginal mucosa, which supported CCR5-tropic but not CXCR4-tropic HIV-1 replication, in contrast to vaginal Langerhans cells (LC). These data suggest that these vaginal mDCs might play an active role in the selection of transmitted viral variants during heterosexual HIV-1 acquisition (54).

In the context of immune control of HIV-1 infection, recent studies suggest that monocytes and mDCs from HIV-1 controllers restrict early HIV-1 replication steps, specifically at the level of viral integration (44, 55, 56) while restriction of viral reverse transcription is less obvious, possibly due to lower induction of SAMHD1 expression in HIV-1 controllers upon exposure to HIV-1. This specific replicative pattern of HIV-1 may enable enhanced cytoplasmic sensing of accumulated HIV-1 reverse transcripts, which represent the primary substrate for innate immune recognition, and facilitate antigen processing and presentation (44, 56). Interestingly, although SAMHD1 is thought to be an interferon inducible gene, DCs and CD4^+^ T cells fail to induce its expression in the presence of type I IFNs (57). Therefore, higher permissiveness of mDCs from controllers to viral reverse transcription may represent a key element for supporting cytoplasmic detection of HIV-1 and for inducing potent cell-intrinsic responses that lead to the effective activation of HIV-1-specific T cells (Figure 1).

Although SAMHD1 is recognized as a critical host factor limiting HIV-1 replication in myeloid cells, alternative SAMHD1-independent restriction mechanisms might also be playing a role in effective immunological control of HIV-1 replication. Among them, recognition of the HIV-1 capside by cyclophilin A (41, 58) and TRIM5 α (59, 60) or endogenous levels of β-catenin (52), could be actively contributing to block HIV-1 replication in myeloid cells. In addition, some studies suggest that HIV-1 could trigger TLR activation in DCs (61). Indeed, activation of MDDCs through TLR4 and TLR3 resulted in inhibition of HIV-1 replication steps in DC, while simultaneously increasing their ability to prime HIV-specific CD8^+^ T cells (62). Therefore, TLR-dependent activation of DC could play a relevant role for inducing highly-functional cellular immune responses against HIV-1. Supporting this idea, polymorphisms in the TLR3 gene confer resistance to HIV-1 infection (63). In fact, it was recently suggested that TLR activation could be playing an active role in the detection of HIV-1 by primary CD141^+^ mDCs (53). Therefore, more studies are required to investigate the mutual interplay between viral restriction in DCs and immune control of HIV-1, and to determine the contribution of myeloid cells to persisting viral reservoirs during suppressive antiretroviral therapy.

## Innate Immune Responses to HIV-1 in DCs

DCs are, in principle, capable of inducing secretion of type I IFNs upon recognition of viral nucleic acids, which subsequently leads to transcription of interferon stimulated genes (ISGs) and the upregulation of class II HLA and costimulatory molecules. As a result of such cell-intrinsic, IFN-dependent immune responses, mature DCs become more restrictive for viral replication, while the expression of molecules involved in antigen presentation and co-stimulation is increased. Whether mDCs can induce secretion of type I IFNs in response to HIV-1 is still highly controversial. In MDDCs, HIV-1 seems to be able to induce expression of several IFN-related genes in the absence of actual production of IFN α/β due to the selective activation of IRF-1 mediated signaling instead of inducing phosphorylation of IRF3, which is known to be required for induction of type I IFNs (64). However, the intracellular DNA sensor cGAS is expressed by myeloid cells (65) and is able of producing cGAMP second messengers upon recognition of HIV-1 DNA (66), leading to the activation of the sensor STING and the signal transducer TBK-1, which promote IFN β production (67, 68). Thus, primary DCs are, in principle, able to sense and induce type I IFN upon exposure to cytoplasmic HIV-1 DNA. In fact, activation of cGAS seems to be required for the transcription of IFN β by primary mDCs and MDDCs in the context of HIV-1 and other viral infections (44, 69, 70). This DNA-dependent mechanism of viral sensing leading to type I IFN responses might be more active in human CD1c^+^ mDCs compared to CD141^+^ mDCs (53). Interestingly, cGAS triggers TLR9-independent activation of primary pDCs in response to intracellular DNA (71, 72). However, current phenotypic markers for pDC identify a heterogenous cell population that, in addition to *bona fide* pDC, contains pre-DC precursors of mDCs, which could also be differentially contributing to the observed responses to HIV-1 (73). Such heterogeneity could be the result of different pre-pDC and/or pDCs originated from either lymphoid or myeloid precursors with different functional properties (74–76). Therefore, more studies are required to elucidate the contribution of TLR-independent sensing of HIV-1 in pDCs. Finally, activation of the cGAS pathway by HIV-1 might involve interactions with additional host factors such as the newly identified NONO protein, which apparently is able to bind cGAS and the HIV-1 capsid and facilitate innate sensing of HIV-1 DNA in dendritic cells (77).

Importantly, preserved or enhanced induction of IFN responses has been described in both primary pDCs (23, 24) and mDCs (44) from HIV-1 controllers exposed to HIV-1. A recent single-cell RNAseq study has identified a highly functional population of CD64^Hi^CD86^Hi^ PD-L1^Hi^ mDCs characterized by a strong type I IFN signature that is induced more efficiently in HIV-1 controllers than in progressors or healthy individuals in response to HIV-1 (78). The induction of such highly functional mDCs depended on the activation of TBK-1, which acts downstream of several intracellular sensing pathways including cGAS and TLR-3. Therefore, enhanced innate recognition of HIV-1 by both pDCs and mDCs might be a contributing factor to develop effective HIV-1 specific immunity in these individuals (Figure 1). However, HIV-1 might have evolved to minimize such mechanisms of viral DNA recognition, since HIV-1 Vif and Vpr are capable of inactivating TBK-1 which is downstream of the cGAS-STING pathway (79). Therefore, additional mechanisms such as alterations in the activation threshold of intracellular sensing pathways might be playing a role in DCs from controllers. In addition to sensing of viral DNA by cGAS, viral immune recognition in DCs could be connected with the RIG-I pathway, which may also contribute to activation of DCs in response to HIV-1 (80). In fact, communication and collaboration between RIG-I and DNA sensing pathways has been reported to amplify innate immune responses against intracellular viral DNA (81, 82). Although no genetic alterations in genes encoding for innate immune sensors for HIV-1 have been found in GWAS studies including large HIV-1-infected populations, a more targeted analysis of innate immune genes may in the future allow to identify immunogenetic polymorphisms in the innate immune system that facilitate innate immune sensing and natural viral control in specific subgroups of HIV-1 controllers.

## Antigen Presenting Cell Function of DCs and Adaptive Immunity Against HIV-1

Given associations between the polyfunctionality of T cell responses and natural progression of HIV-1 infection (8), several studies have focused on the function of DCs as professional antigen presenting cells (APC) and how these cells are involved in the priming of adaptive immune cells. As mentioned before, both mDCs and pDCs can respond and mature to a certain degree in response to HIV-1, but may become exhausted and hyporesponsive during chronic progressive infection, which might impact their antigen-presenting cell function (83). In the case of pDCs, infection with HIV-1 seems to turn these cells more tolerogenic, and increase their potential to drive polarization of CD4^+^ T cells into immunosuppressive T regulatory cells (84). On the other hand, although pDCs can activate CD8^+^ T cells through cross-presentation (85), no studies have yet analyzed the impact of pDCs on the priming of HIV-1-specific cytotoxic CD8^+^ T cell responses. In contrast, while circulating mDCs from healthy individuals are functionally incapable of efficiently priming T cells *in vitro* after exposure to HIV-1 (86), effective antigen presenting functions of mDCs from HIV-1 elite controllers is associated with enhanced abilities to prime HIV-specific CD8^+^ T cells in these patients (44, 78). In addition to mDCs, recent *in vitro* studies have shown that MDDCs can acquire HIV-1 antigens from Langerhans cells, become activated and induce cross-presentation to CD8^+^ T cells (87), suggesting that these cells may also be potentially able to prime protective HIV-1-specific cytotoxic CD8^+^ T cells. In addition, independent studies have shown that MDDCs can mediate cross-presentation of immuno-dominant HIV-1 peptides and activate HIV-1-specific CD8^+^ T cells (88). However, MDDC are not a physiological DC subset present and in fact more closely resemble inflammatory DCs (89). Nevertheless, a recent evaluation suggested that primary CD141^+^ mDCs, obtain HIV-1 antigens from infected CD1c^+^ mDCs for cross-presentation to CD8^+^ T cells (53). Interestingly, DCs infected with HIV-1 can also present endogenous viral peptides and mediate activation of HIV-1-specific CD4^+^ T cells (90). Therefore, mDCs and MDDCs might be involved in the priming of effective HIV-1-specific T cell responses observed in controllers.

Although highly functional HIV-1-specific CD8^+^ T cell responses were identified as the main correlate of antiviral immune defense (91), the discovery of broadly neutralizing antibodies (bNAbs) against multiple strains of HIV-1 (92, 93), has led to a great interest in understanding their potential contribution to spontaneous immunological control of HIV-1. Recent works have indeed identified a subpopulation of HIV-1 viremic controllers who develop bNAbs in the absence of high levels of viremia or immune activation (94). In previous studies in viremic HIV-1-infected progressors, the induction of bNAbs was associated with the presence of CXCR5^+^ PD-1^+^ T follicular helper cells (Tfh) in the blood (95). Although Tfh cells facilitate B cell maturation and immunoglobulin class switching in lymphoid tissue (96), peripheral CXCR5^+^ PD-1^+^ CD4^+^ T lymphocytes have been proposed to act as peripheral counterparts of Tfh cells (pTfh) (95, 97) and could serve as a peripheral biomarker of high germinal center Tfh cell activity. Therefore, the priming of Tfh cells by mDC might be important in HIV-1 controllers capable of inducing antibodies with broader neutralizing activity. Supporting this possibility, mDCs from controller neutralizers are more efficient in priming CD4^+^ T cells into long lived PD-1^Lo^ Tfh precursors, which can differentiate into functional PD-1^Hi^ Tfh effector cells upon antigenic stimulation (98). Importantly, higher frequencies of PD-1^Lo^ Tfh precursors in the blood are correlated with higher breadth of Ab neutralization in this subset of controllers. Compatible with an indirect role of DCs for influencing humoral immunity through polarization of Tfh, mDCs from controller neutralizers are characterized by high levels of CD40, a molecule previously involved in Tfh cell differentiation (99), and display distinct transcriptional patterns that differ from those present in CD64^Hi^ PD-L1^Hi^ mDCs from elite controllers with high CD8^+^ T cell priming potential (98). Therefore, these findings might suggest a range of functional specializations of mDCs from controllers that may contribute to immune viral control through different immune mechanisms. To which degree individual DC subpopulations influence other components of the innate and adaptive immune system and contribute to control of HIV-1 is still an open question that requires further study.

## Conclusions

In this review, we have summarized recent advances in understanding DC biology in the context of HIV-1 immune control. While studies have revealed multiple mechanisms by which DCs might contribute to controlling HIV-1 (Figure 1), future studies will be necessary to evaluate the complexity of individual DC subsets in promoting beneficial versus detrimental effects during HIV-1 infection. Similarly, our knowledge about the intrinsic ability of pDCs and mDCs to sense and respond to HIV-1 has greatly improved over the last few years, but translating this insight into improved and more specific adjuvants for future preventive and therapeutic HIV-1 vaccines represents a considerable challenge. The development to new humanized animal models that recapitulate human DC biology will likely be critical to identify effective vaccination strategies based on DCs. Together, DCs are emerging as critical players of effective immune responses in HIV-1 and a closer understanding of these cells might contribute to the development of novel effective vaccines or immunotherapies.

## Author Contributions

EM-G and XY conceived and wrote the manuscript.

### Conflict of Interest Statement

The authors declare that the research was conducted in the absence of any commercial or financial relationships that could be construed as a potential conflict of interest.
